# The Impact of Decaffeinated Green Tea Extract on Fat Oxidation, Body Composition and Cardio-Metabolic Health in Overweight, Recreationally Active Individuals

**DOI:** 10.3390/nu13030764

**Published:** 2021-02-26

**Authors:** Justin D. Roberts, Ashley G. B. Willmott, Liam Beasley, Mariette Boal, Rory Davies, Laurence Martin, Havovi Chichger, Lata Gautam, Juan Del Coso

**Affiliations:** 1 Cambridge Centre for Sport and Exercise Sciences, School of Psychology and Sport Science, Anglia Ruskin University, Cambridge CB1 1PT, UK; ash.willmott@aru.ac.uk (A.G.B.W.); marietteboal.student@gmail.com (M.B.); rorydavies.student@gmail.com (R.D.); lorymartin.student@gmail.com (L.M.); 2 Department for Health, University of Bath, Bath BA2 7AY, UK; lab90@bath.ac.uk; 3 School of Life Sciences, Anglia Ruskin University, Cambridge CB1 1PT, UK; havovi.chichger@aru.ac.uk (H.C.); lata.gautam@aru.ac.uk (L.G.); 4 Centre for Sport Studies, Rey Juan Carlos University, 28943, Fuenlabrada, Spain; juan.delcoso@urjc.es

**Keywords:** green tea extract, fat oxidation, body fat, weight loss, exercise

## Abstract

This study investigated the effect of decaffeinated green tea extract (dGTE), with or without antioxidant nutrients, on fat oxidation, body composition and cardio-metabolic health measures in overweight individuals engaged in regular exercise. Twenty-seven participants (20 females, 7 males; body mass: 77.5 ± 10.5 kg; body mass index: 27.4 ± 3.0 kg·m^2^; peak oxygen uptake ($\dot{V}$O_2peak_): 30.2 ± 5.8 mL·kg^−1^·min^−1^) were randomly assigned, in a double-blinded manner, either: dGTE (400 mg·d^−1^ (−)-epigallocatechin−3-gallate (EGCG), *n* = 9); a novel dGTE+ (400 mg·d^−1^ EGCG, quercetin (50 mg·d^−1^) and α-lipoic acid (LA, 150 mg·d^−1^), *n* = 9); or placebo (PL, *n* = 9) for 8 weeks, whilst maintaining standardised, aerobic exercise. Fat oxidation (‘FAT_MAX_’ and steady state exercise protocols), body composition, cardio-metabolic and blood measures (serum glucose, insulin, leptin, adiponectin, glycerol, free fatty acids, total cholesterol, high [HDL-c] and low-density lipoprotein cholesterol [LDL-c], triglycerides, liver enzymes and bilirubin) were assessed at baseline, week 4 and 8. Following 8 weeks of dGTE+, maximal fat oxidation (MFO) significantly improved from 154.4 ± 20.6 to 224.6 ± 23.2 mg·min^−1^ (*p* = 0.009), along with a 22.5% increase in the exercise intensity at which fat oxidation was deemed negligible (FAT_MIN_; 67.6 ± 3.6% $\dot{V}$O_2peak_, *p* = 0.003). Steady state exercise substrate utilisation also improved for dGTE+ only, with respiratory exchange ratio reducing from 0.94 ± 0.01 at week 4, to 0.89 ± 0.01 at week 8 (*p* = 0.004). This corresponded with a significant increase in the contribution of fat to energy expenditure for dGTE+ from 21.0 ± 4.1% at week 4, to 34.6 ± 4.7% at week 8 (*p* = 0.006). LDL-c was also lower (normalised fold change of −0.09 ± 0.06) for dGTE+ by week 8 (*p* = 0.038). No other significant effects were found in any group. Eight weeks of dGTE+ improved MFO and substrate utilisation during exercise, and lowered LDL-c. However, body composition and cardio-metabolic markers in healthy, overweight individuals who maintained regular physical activity were largely unaffected by dGTE.

## 1. Introduction

Green tea (GT) polyphenols have been widely investigated for their potential therapeutic health benefits from an antioxidant [1], anti-inflammatory [2,3], chemoprotective [4] and cardio-metabolic perspective [5,6,7]. The catechin content of GT, of which (−)-epigallocatechin−3-gallate (EGCG) may account for up to 80% [8], may be primarily related to adaptive mechanisms pertinent to health benefits. GT catechins may have specific thermogenic effects through the inhibition of catechol-o-methyl transferase (COMT) and subsequent catecholamine, cyclic adenosine monophosphate (cAMP) and lipolytic activity [9]. Consequently, these effects may enhance whole-body fat oxidation [10,11], and GT catechins may have important metabolic effects pertinent to reduced adipose tissue [12], improved body mass index (BMI) and/or body composition [10], and lowered circulating metabolites (e.g., low-density lipoprotein cholesterol (LDL-c) [13]). However, such mechanisms have also been challenged based on limited evidence from studies [14]. Regular consumption of GT catechins has also been proposed to exert ‘calorie-restriction-mimetic’ effects [15] over time, through modulated cell signalling (e.g., peroxisome proliferator-activated receptor gamma coactivator 1-alpha (PGC1-α), sirtuin 1 (SIRT1) and AMP-activated protein kinase (AMPK) pathways), influencing gene expression associated with mitochondrial efficiency and fat oxidation [14], and is supported through emerging experimental evidence [16].

In terms of fat oxidation, studies with administration of GT extract (GTE) for <7 days, providing between 270 and 375 mg·d^−1^ EGCG, have reported significant improvements in whole-body fat oxidation rates in untrained and overweight individuals both at rest [17,18], during exercise [18] and post-exercise [19], as well as increased 24 hour energy expenditure [11]. Elevated catecholamine and glycerol concentrations in response to acute EGCG supplementation [11,19] support mechanisms associated with COMT inhibition. However, this may, in part, be explained through the inclusion of high-intensity exercise invoking elevated catecholamine levels [19]. Surprisingly, the benefits observed in these studies occurred despite GTE consumption with food (which has been reported to reduce GTE bioavailability [20]), inferring that observed improvements in fat oxidation likely occurred in response to pre-test fasted intake of GTE. Elsewhere, studies have reported no effect of short-term GTE supplementation on fat oxidation in trained individuals, with doses between 270 and 506 mg·d^−1^ EGCG [21,22], suggesting that GTE may be beneficial only for untrained/overweight individuals.

In the longer term (>3 weeks), the effects of GTE supplementation on fat oxidation are equivocal. Low-dose GTE supplementation (68 mg·d^−1^ EGCG) for 3 weeks did not improve fat oxidation during exercise in trained individuals [23,24], although improvements in cardio-metabolic and inflammatory metabolites (e.g., increased high-density lipoprotein cholesterol (HDL-c) [23], and reduced C-reactive protein [24]) were reported. A four-week supplementation period of moderate-dose decaffeinated GTE (dGTE; 400 mg·d^−1^ EGCG) significantly improved total fat oxidation rates during exercise, and reduced body fat percentage in one study [25], but not elsewhere, even when employing higher-dose dGTE (624 mg·d^−1^ EGCG [26]), with both studies involving healthy, lean males.

With regards to cardio-metabolic risk factors, studies lasting 8-12 weeks also provide equivocal findings. Low-dose GTE supplementation (99 mg·d^−1^ GTE) for 8 weeks was not effective in improving body fat percentage, BMI, blood pressure, triglyceride and LDL-c levels over that obtained through introduction of aerobic training in overweight/obese, previously sedentary females [27]. Likewise, high-dose caffeinated GTE (1206.9 mg·d^−1^ GTE; 595.8 mg·d^−1^ EGCG) for 12 weeks did not improve resting energy expenditure, body mass, BMI, waist to hip ratio (WHR) or fat mass over that explained through sustained inclusion of a low-energy diet in overweight woman [28]. In contrast, an eight-week GTE supplementation period (500 mg·d^−1^ GTE; ~225 mg·d^−1^ EGCG) significantly improved body mass, BMI, body fat percentage, visceral fat area (VFA) and adiponectin levels above that obtained through introduction of aerobic training in previously sedentary overweight women [29] and men [30]. Therefore, it is unclear whether GTE supplementation positively impacts on fat oxidation, body composition and cardio-metabolic risk factors in overweight individuals, particularly when unaccustomed diet or exercise interventions are introduced.

In addition to the range of EGCG doses used, confounding factors such as GTE type and bioavailability may, in part, explain the varying findings observed. Only a handful of studies have employed a dGTE formula [17,18,25,26], whereas positive benefits found elsewhere could be explained through the thermogenic effects of caffeine [11,19,29,30]. Whilst positive findings have been observed with a moderate EGCG dose (>300 mg·d^−1^), improving the bioavailability and effectiveness of GTE through standardised consumption away from food [20] and/or inclusion of antioxidant nutrients (e.g., quercetin, α-lipoic acid (LA), curcumin [31,32,33,34]) may lead to enhanced fat oxidation, body composition, and/or cardio-metabolic risk factor improvement, particularly in overweight individuals. Indeed, precedent for the approach of combining GTE with quercetin or LA has previously been observed in animal models [35,36]. Elsewhere, use of a GT beverage with antioxidant supplementation has been shown to enhance exercise capacity, glucose tolerance and body composition in older individuals [37]. However, there is a paucity of research confirming potential benefits of GTE compounds with human participants, despite novel combinations already being commercially available. Therefore, it is pertinent to investigate the efficacy such compounds compared with GTE only use.

Therefore, this study aimed to assess the longer-term impact of moderate dGTE supplementation (with or without antioxidant nutrients) on fat oxidation, body composition and cardio-metabolic risk factors in overweight, but healthy individuals engaged in regular exercise. It was hypothesised that fat oxidation would be improved with dGTE (particularly when additional antioxidant nutrients are included), resulting in improved body composition and cardio-metabolic risk factors compared with placebo.

## 2. Materials and Methods 

### 2.1. Ethical Approval and Study Participants

This study was conducted in accordance with the Declaration of Helsinki (2013), and registered with ClinicalTrials.gov (Identifier: NCT04628624). Ethical approval was obtained from the Faculty of Science and Technology Research Ethics Panel, Anglia Ruskin University (FST/FREP/17/703). Following a priori power calculation assessment (G*power3, Dusseldorf, Germany [38]; using α = 0.05; 1 − β = 0.80; based on observed fat oxidation data [18,25]), a minimum sample size of 7 (per group) was estimated. A total cohort of 44 participants volunteered for pre-screening selection, from which 13 participants did not meet the study inclusion criteria. 

Thirty-one participants were therefore invited to take part in the intervention study. However, 2 participants withdrew at week 4 for personal reasons, and data for 2 participants were excluded based on not meeting supplement adherence or testing criteria. Therefore, a total of 27 participants (20 females, 7 males; mean ± standard deviation (SD), age: 43 ± 8 years; body mass: 77.5 ± 10.0 kg; height: 1.68 ± 0.06 m; BMI: 27.4 ± 3.0 kg·m^2^; initial relative peak oxygen uptake $(\dot{V}$O_2peak_): 30.2 ± 5.8 mL·kg^−1^·min^−1^) satisfactorily completed the intervention, with study power deemed sufficient based on a priori analysis. Participant characteristics for each intervention group are displayed in Table 1. All participants provided written, informed consent, and satisfactorily completed a general health screen prior to study inclusion.

### 2.2. Study Design and Eligibility

This study employed a randomised, repeated-measures, double-blinded, placebo-controlled design over an 8 week period. Following a full study briefing and provision of written, informed consent, volunteers took part in an initial pre-screening assessment with full familiarisation to the main study testing procedures outlined below. Study eligibility required volunteers to: satisfactorily complete a general health screen questionnaire; be over 18 years of age and ‘recreationally active’ (defined as general exercise activity 1–3 times per week) with a BMI between 25.0 and 29.9 kg·m^2^ and/or a body fat percentage in the overweight category for age/gender based on guidelines set out by the American College of Sports Medicine (ACSM) [39]. As a means to further quantify ‘recreationally active’, participants were required to have a relative $\dot{V}$O_2peak_ between 20 and 45 mL·kg^−1^·min^−1^ during pre-screening testing. Participants with a known history of cardio-metabolic disorders, blood- and liver-related disorders, and recent viral infections were not eligible for study inclusion. Participants were also required to not be consuming other nutritional or thermogenic supplements (e.g., GTE, creatine, weight loss products) or medication for at least 4 weeks prior to beginning the study intervention.

### 2.3. Laboratory Procedures

All testing procedures were carried out in the Cambridge Centre for Sport and Exercise Sciences, Human Physiology Laboratory, Anglia Ruskin University under controlled environmental conditions (M ± SD; temperature: 18.5 ± 1.3 °C; barometric pressure: 1009.4 ± 12.2 mBar; and relative humidity: 44.7 ± 7.8%). In addition to initial pre-screening, participants visited the laboratory at baseline (week 0), and at the end of weeks 4 and 8 of the intervention period. All testing was undertaken in the morning, with participants being tested at the same time of day across the intervention. In the week leading into each laboratory visit, participants were required to complete dietary intake and activity diaries (see Section 2.5). In addition, as a means to standardise each pre-testing period, in the 24 hours prior, participants were required to rest/no exercise, consume a standardised evening meal (~10 kcal·kg^−1^, of which 50% carbohydrate, 20% protein, 30% fat), and arrive in a euhydrated, fasted state (~10 hours) having refrained from consuming caffeinated products prior to testing. 

Upon arrival, participants rested for ~10 min in a supine position prior to duplicate assessment of resting heart rate and blood pressure (Omron 705CP, Kyoto, Japan). Following this, a venous whole blood sample was collected from participants by a qualified phlebotomist into triplicate 4 mL serum clot activator Vacuette™ tubes (Greiner Bio-One GmbH, Kremsmunster, Austria). Samples were centrifuged for 10 min at 2000 rcf according to manufacturer recommendations, with aliquotted serum pipetted into sterile, nonpyrogenic, polypropylene cryovials (Fisherbrand, Fisher Scientific, Loughborough, UK) and frozen at −80 °C for later assessment of blood analytes (see Section 2.6).

#### 2.3.1. Anthropometric Measures

Participants then underwent body composition assessment, with height measured using a stadiometer (Seca CE123, Hamburg, Germany). Body mass and body fat percentage were assessed through the use of bioelectrical impedance analysis scales (Tanita SC-330ST, Amsterdam, The Netherlands). Body fat percentage was additionally confirmed using an 8-site skinfold calliper assessment (following guidelines outlined by the International Society for the Advancement of Kinanthropometry (ISAK)). Specific girth measures were undertaken for abdomen, thigh and calf locations, as well as waist to hip ratio (WHR) conforming to ISAK/ACSM guidelines. Sagittal abdominal diameter (SAD) was also measured using a Holtain-Kahn abdominal caliper (model 609, Holtain Ltd., Crosswell, Pembrokeshire, UK), with visceral fat area (VFA) estimated from validated equations [40]. Anthropometric measures were undertaken in triplicate by the same researcher across the study period, with average readings for each measure used for analyses.

#### 2.3.2. Incremental Exercise Fat Oxidation Assessment

Participants were fitted with a Polar FS2 telemetric heart rate (HR) monitor (Polar Electro Ltd., Kempele, Finland) and a suitable face-mask for expired air assessment (Hans Rudolph 7450, Cranlea Human Performance Ltd., Bournville, Birmingham, UK) prior to an initial resting period. Resting expired air was then measured using a Metalyser 3B automated gas-analyser (Cortex Biophysik, Leipzig, Germany), after which participants underwent a maximal fat oxidation (‘FAT_MAX_’) test using a Lode Excalibur sport cycle-ergometer (Lode B.V., Groningen, The Netherlands). The FAT_MAX_ protocol was based on original research [41], but modified in line with recent studies involving similar cohorts to the present study [42] and pilot work assessment. 

Briefly, participants completed a graded exercise test to volitional exhaustion, starting at 30 W (acting as a warm-up stage) with 20 W increments every 3 min. Cadence was maintained between 75 and 85 rpm throughout. Breath-to-breath expired air analysis was undertaken throughout the test, along with telemetric HR and self-assessed rating of perceived exertion (RPE) [43] in the last minute of each stage. Maximal individual effort was determined when two of the following criteria were met: (i) a change in oxygen consumption ($\dot{V}$O_2_) of <2 mL·kg^−1^·min^−1^ with increasing workload; (ii) attainment of 95% of age-predicted HR_max_ (220-age); (iii) a respiratory exchange ratio (RER) > 1.05; and (iv) an RPE >9 (0–10 scale) along with evidence of volitional fatigue symptoms. $\dot{V}$O_2peak_ was estimated from the highest 30 sec moving average oxygen consumption.

Fat oxidation rates (FAT_OX_; mg·min^−1^) were derived from $\dot{V}$O_2_ and carbon dioxide ($\dot{V}$CO_2_) expired air assessment (L·min^−1^) using stoichiometric equations [18,44], with protein oxidation assumed negligible, as follows:

(1) $${\mathrm{FAT}}_{\mathrm{OX}}=(1.695\;\times \;\dot{V}O_{2})\;-\;(1.701\;\times \;\dot{V}CO_{2}),$$

Using individual FAT_OX_ data for each stage plotted against exercise intensity (% $\dot{V}$O_2peak_), a 2nd-order polynomial curve was fitted from which maximal fat oxidation rate (MFO; mg·min^−1^), maximal fat oxidation exercise intensity (FAT_MAX_, defined as % $\dot{V}$O_2peak_ at which MFO occurred) and minimum fat oxidation exercise intensity (FAT_MIN_, defined as the % $\dot{V}$O_2peak_ where FAT_OX_ was deemed zero) were derived, based on previous research [45].

#### 2.3.3. Steady State Exercise Assessment

Following a standardised 30 min rest period (with resting $\dot{V}$O_2_ and HR comparable to pre-exercise conditions), participants underwent a fixed-intensity exercise test (60% of individual $\dot{V}$O_2peak_), using the same cycle-ergometer, with cadence maintained between 75 and 85 rpm for 60 min. Perceived exertion and HR were measured at 5 min and each 15 min thereafter. Breath-to-breath expired air was continuously measured throughout the submaximal exercise period, with 60 sec average data recorded upon test completion. Fat oxidation rates were derived from the previous stoichiometric Equation (1). Carbohydrate oxidation rates (CHO_OX_; mg·min^−1^) and energy expenditure (EE; kcal·min^−1^) were derived from the following equations [44]:

(2) $${\mathrm{CHO}}_{\mathrm{OX}}=(4.210\;\times \;\dot{V}{\mathrm{CO}}_{2})\;-\;(2.962\;\times \;\dot{V}O_{2}),$$

(3) $$\mathrm{EE}=(0.550\;\times \;\dot{V}{\mathrm{CO}}_{2})\;+\;(4.471\;\times \;\dot{V}O_{2}),$$

Additionally, both fat and carbohydrate oxidation stability (expressed as a %) were assessed on the basis of the duration maintained within ±10% of mean FAT_OX_ and CHO_OX_ rates, respectively. Contributions of both carbohydrate and fat to EE were calculated on the basis of caloric conversion of estimated total CHO_OX_ and FAT_OX_ (g·h^−1^) expressed as a percentage of EE (kcal·h^−1^).

### 2.4. Nutritional Interventions

Following baseline assessment, participants were allocated, in a double-blinded manner, to one of three intervention groups using a random number generator (www.randomizer.org accessed on 26 February 2021). Participants were randomly assigned to an 8 week intervention (2 hydroxypropyl methyl cellulose capsules daily, split dose taken away from food) of either: (i) placebo (PL; containing a total of 892 mg·d^−1^ potato fibre as a bulking agent); (ii) decaffeinated green tea extract (dGTE; 580 mg·d^−1^ dGTE, 70% standardised, delivering 400 mg·d^−1^ EGCG, matched weighted with additional bulking agent, negligible caffeine trace (≤0.5%)); or (iii) a novel decaffeinated green tea extract formula (dGTE+; 580 mg·d^−1^ dGTE (400 mg·d^−1^ EGCG, negligible caffeine trace ≤0.5%), with quercetin (50 mg·d^−1^) and α-lipoic acid (LA; 150 mg·d^−1^), also matched weighted with additional bulking agent). 

All supplementation was supplied and pre-capsulated by Biocare Ltd. (Redditch, Birmingham, UK), and verified through Nutraceuticals Group Europe (Merstham, Surrey, UK) with dGTE sourced from Changsha Active Ingredients Group Inc. (Changsha, China), based on our previous research [25]. All supplements were provided in standardised opaque sealed pots for hygiene and double-blinding purposes. To monitor supplement adherence, participants completed a daily compliance record throughout the intervention. As a further measure, participants were initially provided a 4 week supplement supply and returned pots at the first follow-up session, with excess capsules counted. Participants were then provided with a second 4 week supply, with pots returned and checked at the final testing session, and overall adherence levels cross-referenced against compliance records. A threshold target of 90% adherence was used for protocol compliance.

### 2.5. Dietary Intake and Exercise Monitoring

Following the pre-screening session, participants included in the main intervention completed a 7 day food diary using a smart phone application (www.MyFitnessPal.com accessed on 26 February 2021), validated in previous research [46,47]. This served partly as a familiarisation process, and also to gain awareness of typical individual dietary intakes. Throughout this study, participants were requested to maintain typical dietary patterns for consistency. Furthermore, in the 7 day period leading into each laboratory session, participants completed a habitual record of dietary intake to assess for consistency. Individual guidance was provided in diary collation, with emphasis on meal content, portion size and weight, and fluid intake. Diaries were assessed using Nutritics Professional Dietary Analysis software (Nutritics Ltd., Co. Dublin, Ireland) by the same researcher. In addition, individual food items were cross referenced against the U.S. Department of Agriculture (USDA) database on the flavonoid content of selected foods, and the Phenol-Explorer database (www.phenol-explorer.eu accessed on 26 February 2021) as a means to estimate dietary quercetin intake.

In conjunction with this, participants were also provided with a standard activity log and HR monitor, and requested to track all exercise activity prior to the start of the intervention (acting as habitual reference), and each week across the 8 week study period. Participants recorded exercise activity, mean session HR, duration and overall session rating of perceived exertion (sRPE) from which training load, monotony and strain were determined as previously described [48,49]. To standardise weekly physical activity, and aligned with participants’ habitual exercise patterns, following the baseline laboratory session, participants were provided with general exercise guidance to be maintained throughout this study. This involved targeting ~150 min of moderate aerobic exercise (~3–5 sessions·wk^−1^), at ~60–65% $\dot{V}$O_2peak_ (~75–80%HR_max_) in accordance with guidelines set out by the American College of Sports Medicine [39,50].

### 2.6. Biochemical Assays

All blood samples were analysed independently in conjunction with the NIHR Cambridge Biomedical Research Centre, Core Biochemical Analysis Laboratory (CBAL), Addenbrookes Hospital, Cambridge. Serum samples were assessed at week 0, 4 and 8 using a Siemens Dimension EXL analyser (Siemens Healthcare Ltd., Camberley, Surrey, UK) for the following analytes (between-batch imprecision (BBI) included): glucose (BBI: 3.6% at 3.5 mmol·L^−1^); triglycerides (Tg; BBI: 5.5% at 0.9 mmol·L^−1^, 3.4% at 2.4 mmol·L^−1^); total cholesterol (Tc; BBI: 4.9% at 2.5 mmol·L^−1^, 2.0% at 6.2 mmol·L^−1^); and HDL-c (BBI: 4.6% at 0.9 mmol·L^−1^, 3.6% at 2.3 mmol·L^−1^), with LDL-c (BBI: 3.9% at 1.4 mmol·L^−1^, 5.0% at 3.2 mmol·L^−1^) derived from the Friedewald equation [51] as follows:

LDL-c = Tc − HDL-c − (Tg/2.2),(4)

Additionally, serum insulin was assessed using a sandwich chemiluminescence immunoassay via a DiaSorin Liaison XL analyser (DiaSorin, Saluggia (VC), Italy), with BBI noted as 11.0% at 34.0 pmol·L^−1^ and 7.0% at 135.0 pmol·L^−1^. An updated Homeostasis Model Assessment (HOMA) was employed to estimate steady state beta cell function (%B), insulin sensitivity (%S) and insulin resistance (IR) using a HOMA2 calculator (v2.2.3., Diabetes Trials Unit, University of Oxford, UK) based on previous research [52]. Serum glycerol was quantified using a coupled enzyme assay involving glycerol kinase and glycerol phosphate oxidase (Sigma-Aldrich, Merck Life Science UK Ltd., Gillingham, Dorset, UK; no BBI quoted). Based on the enzymatic conversion of free fatty acids (FFA) to acyl CoA by acyl-Co A synthetase, FFA were determined using a Roche half-micro test assay kit (Sigma-Aldrich, Merck Life Science UK Ltd., Gillingham, Dorset, UK; BBI: 12.5% at 122 µmol·L^−1^, 4.5% at 466 µmol·L^−1^). Serum leptin and adiponectin were assessed using an AutoDELFIA analyser (Perkin Elmer LAS (UK) Ltd., Beaconsfield, Buckinghamshire, UK), employing in-house two-site microtiter plate-based DELFIA assay (BBI for leptin: 7.1% at 2.7 ng·ml^−1^, 3.9% at 14.9 µg·ml^−1^ and 5.7% at 54.9 µg·ml^−1^; BBI for adiponectin: 5.4% at 3.6 µg·ml^−1^, 5.2% at 9.2 µg·ml^−1^ and 5.8% at 15.5 µg·ml^−1^), with antibodies and standards supplied by R&D systems (R&D Systems Europe, BioTechne, Abingdon, UK). 

An important feature of the current study, and with recent concerns around potential for high-dose catechin-related hepatic toxicity, a liver enzyme panel was also carried out using a Siemens Dimension EXL analyser (Siemens Healthcare Ltd., Camberley, Surrey, UK) for the following analytes: alkaline phosphatase (ALP; BBI: 16.3% at 40 U·L^−1^, 6.4% at 282 U·L^−1^); alanine aminotransferase (ALT; BBI: 8.1% at 22 U·L^−1^, 3.6% at 180 U·L^−1^); aspartate aminotransferase (AST; BBI: 5.0% at 38 U·L^−1^, 2.5% at 253 U·L^−1^); and bilirubin (BBI: 2.7% at 14 µmol·L^−1^, 1.0% at 74 µmol·L^−1^). As a means to detect any acute elevations in liver enzymes, participants also had an additional blood sample collected at week 2 of the intervention as a precautionary measure. 

### 2.7. Statistical Analyses

Statistical analyses were performed using SPSS (IBM, Version 26.0). Normality of data was verified by the Shapiro–Wilk test. Outliers were identified by inspection of box plots > 1.5 interquartile range in SPSS. Baseline measures were assessed using a between-groups analysis of variance (ANOVA), or where applicable (e.g., dietary analyses) an independent samples t-test was employed. A mixed-design repeated-measures ANOVA (group, time) were performed for main analyses, with Bonferroni post-hoc comparisons where applicable. Where sphericity was violated a Greenhouse–Geisser correction was applied. An alpha level of ≤0.05 was employed for statistical significance, with effect size (partial eta squared; η_p_^2^) also reported (small = 0.02, medium= 0.13, large = 0.26). Data are reported as the mean ± SE.

## 3. Results

### 3.1. Dietary Intake, Supplement Adherence and Exercise Monitoring

Dietary analysis comparisons across the intervention are shown in Table 2. No significant differences were reported between or within groups across the 8 week intervention period for energy, carbohydrate, fat or protein intake (*p* > 0.05). In addition, no differences were reported between mean energy intake compared with expected maintenance caloric intake (*p* > 0.05) overall or within groups at week 4 or 8, demonstrating dietary consistency.

Across the 8 week intervention, average daily energy intake for dGTE+ was 1918 ± 66 kcal·d^−1^ (or 25.7 ± 1.2 kcal·kg^−1^·day^−1^) compared with 1882 ± 96 kcal·d^−1^ (or 25.6 ± 1.2 kcal·kg^−1^·day^−1^) for dGTE, and 1975 ± 96 kcal·d^−1^ (or 24.3 ± 1.5 kcal·kg^−1^·day^−1^) for PL. Macronutrient intake was also comparable, with an average fat intake of 1.0 ± 0.1 g·kg^−1^·day^−1^ for dGTE+ compared 0.9 ± 0.1 g·kg^−1^·day^−1^ for dGTE and 0.9 ± 0.1 g·kg^−1^·day^−1^ for PL. Likewise, average carbohydrate intake was 2.7 ± 0.2 g·kg^−1^·day^−1^ for dGTE+ compared with 3.0 ± 0.2 g·kg^−1^·day^−1^ for dGTE and 2.7 ± 0.2 g·kg^−1^·day^−1^ for PL. Similarly, average protein intake was comparable between groups at 1.1 ± 0.1 g·kg^−1^·day^−1^ for dGTE+, 1.2 ± 0.1 g·kg^−1^·day^−1^ for dGTE, and 0.9 ± 0.0 g·kg^−1^·day^−1^ for PL. 

Estimation of dietary quercetin highlighted a mean overall intake of 20.6 ± 8.5 mg·d^−1^, with no differences reported between groups (20.1 ± 7.3 mg·d^−1^ for PL; 20.0 ± 8.8 mg·d^−1^ for dGTE; and 21.8 ± 10.4 mg·d^−1^ for dGTE+, *p* > 0.05). Mean adherence to the supplement intervention was 95.4 ± 1.0%. Within groups, adherence rates were 93.0 ± 1.1%, 96.3 ± 0.8% and 96.9 ± 1.3% for PL, dGTE and dGTE+, respectively, with a significant difference reported between PL and dGTE+ (*p* = 0.04) only.

Exercise monitoring training load comparisons are shown in Table 3. No significant differences were reported between groups across the intervention for training load, monotony or strain (*p* > 0.05) demonstrating relative consistency. Mean weekly duration was reported at 189.7 ± 22.4 min, 204.0 ± 20.9 min and 196.4 ± 29.1 min for PL, dGTE and dGTE+, respectively (*p* > 0.05), meeting expected recommendations. No significant differences were observed for mean HR compared to training recommendations (75–80%HR_max_) for PL (82 ± 2%HR_max_), dGTE (79 ± 2%HR_max_) and dGTE+ (79 ± 1%HR_max_), respectively (*p* > 0.05).

### 3.2. Fat Oxidation Data

**Figure 1:**
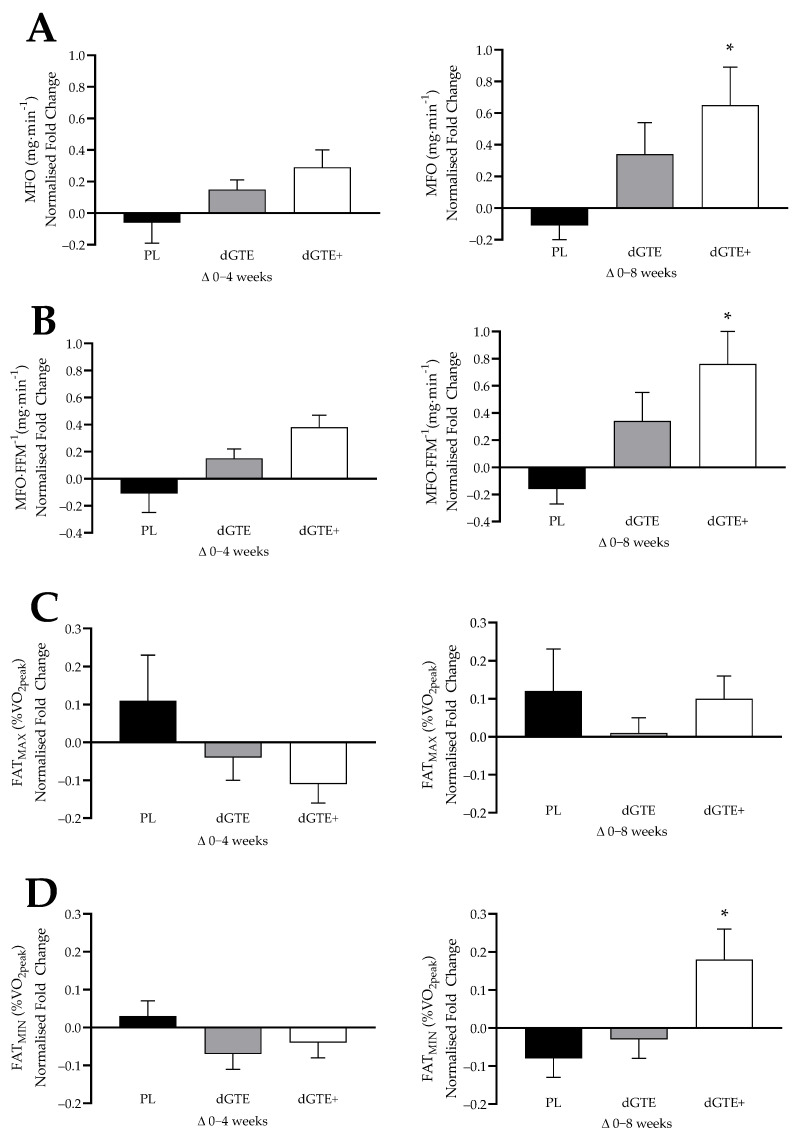
Mean normalised fold change for FAT_MAX_ test parameters between groups by week 4 (Δ0–4 weeks) and week 8 (Δ0–8 weeks) of the intervention; showing (**A**) maximal fat oxidation rate (MFO), (**B**) relative maximal fat oxidation rate per kg FFM (MFO·FFM^−1^), (**C**) exercise intensity at which MFO occurs (FAT_MAX_), and (**D**) exercise intensity where fat oxidation deemed zero (FAT_MIN_). PL = placebo; dGTE = decaffeinated green tea extract; dGTE+ = decaffeinated green tea extract with antioxidants. * significantly different from PL (*p* ≤ 0.034).

Mean fat oxidation measures are shown in Table 4, with normalised fold change shown in Figure 1. No significant differences were reported at baseline for all variables (*p* > 0.05). A significant interaction effect was found for maximal fat oxidation (MFO; F = 3.41, *p* = 0.016, η_p_^2^ = 0.22), with post-hoc analysis highlighting a 45.4% increase in MFO for dGTE+ at week 8 (*p* = 0.009) compared to baseline only. This was supported with a noted trend at week 4 for dGTE+ (*p* = 0.063) also compared to baseline. Similarly, dGTE+ resulted in 49.4% increase in relative MFO (interaction effect; F = 3.67, *p* = 0.011, η_p_^2^ = 0.23) by week 8 compared to baseline (*p* = 0.024), with a trend noted compared to week 4 (*p* = 0.059). dGTE had a positive, but non-significant impact on MFO (+12.7%) and MFO·FFM^−1^ (+13.9%), whereas PL resulted in a non-significant reduction in MFO (−11.8%) and MFO·FFM^−1^ (−21.4%; *p* > 0.05). 

In terms of normalised fold change (Figure 1), a significant effect for MFO was reported overall by week 8 (F = 3.81, *p* = 0.037, η_p_^2^ = 0.24), but not by week 4 (*p* = 0.113). Normalised MFO was significantly greater with dGTE+ only (+0.65 ± 0.25 fold change) compared with PL (−0.11 ± 0.09 fold change; *p* = 0.034) across the intervention. Similarly, normalised relative MFO was significantly different between groups by week 8 (F = 4.16, *p* = 0.028, η_p_^2^ = 0.26) but not by week 4 (*p* = 0.073), again with a highlighted increase for dGTE+ (+0.76 ± 0.24 fold change) compared with PL (−0.16 ± 0.11 fold change; *p* = 0.026). 

No significant effects were found for FAT_MAX_ or HR at FAT_MAX_ within or between groups (*p* > 0.05). However, a positive interaction effect was observed for FAT_MIN_ (F = 6.02, *p* = 0.001, η_p_^2^ = 0.33), with dGTE+ resulting in a 22.5% increase in FAT_MIN_ by week 8 compared with week 4 (*p* = 0.003) and a trend noted compared to baseline (*p* = 0.056). When expressed as normalised fold change, a significant effect was found for FAT_MIN_ by week 8 (F = 4.77, *p* = 0.018, η_p_^2^ = 0.28), with dGTE+ demonstrating a +0.18 ± 0.08 fold change compared with PL (-0.08 ± 0.05; *p* = 0.023). Throughout the intervention, as expected, $\dot{V}$O_2peak_ was maintained for PL: 2.5 ± 0.2 L·min^−1^, 2.4 ± 0.1 L·min^−1^ and 2.4 ± 0.1 L·min^−1^; dGTE: 2.4 ± 0.2 L·min^−1^, 2.5 ± 0.2 L·min^−1^, and 2.4 ± 0.2 L·min^−1^; and dGTE+: 2.1 ± 0.2 L·min^−1^, 2.3 ± 0.2 L·min^−1^, and 2.1 ± 0.2 L·min^−1^, with no differences reported within or between groups at week 0, 4 and 8, respectively (*p* > 0.05).

### 3.3. Steady State Exercise Data

**Figure 2:**
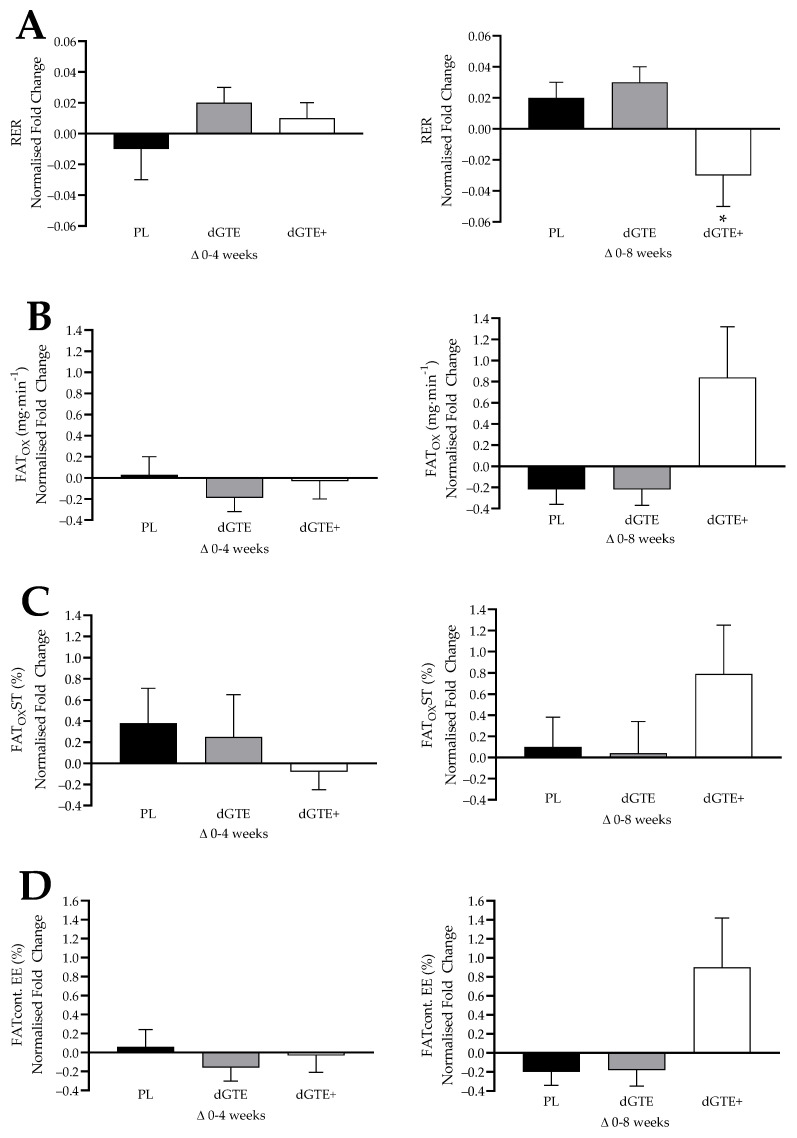
Mean normalised fold change for fat oxidation parameters during steady state exercise between groups by week 4 (Δ0–4 weeks) and week 8 (Δ0–8 weeks) of the intervention; showing (**A**) respiratory exchange ratio (RER), (**B**) fat oxidation rate (FAT_OX_), (**C**) fat oxidation stability (FAT_OX_ST), and (**D**) contribution of fat to total energy expenditure (FATcont.EE). PL = placebo; dGTE = decaffeinated green tea extract; dGTE+ = decaffeinated green tea extract with antioxidants. * significantly different to dGTE only (*p* = 0.034).

Mean steady state exercise measures are shown in Table 5, with normalised fat oxidation data shown in Figure 2. Mean exercise intensity during laboratory testing was comparable across the intervention (baseline: 60.2 ± 1.4% $\dot{V}$O_2peak_, $\dot{V}$O_2_: 1.4 ± 0.1 L·min^−1^; week 4: 59.8 ± 1.6% $\dot{V}$O_2peak_, $\dot{V}$O_2_: 1.4 ± 0.1 L·min^−1^; week 8: 58.7 ± 1.5% $\dot{V}$O_2peak_,$\dot{V}$O_2_: 1.4 ± 0.1 L·min^−1^; *p* > 0.05), with no differences reported within or between groups, or compared to set exercise intensity (*p* > 0.05). No significant differences were reported at baseline for all variables (*p* > 0.05). A significant interaction effect was found for mean RER (F = 4.66, *p* = 0.003, η_p_^2^ = 0.28), with post-hoc analyses revealing a reduction in RER by week 8 for dGTE+ compared with week 4 (*p* = 0.004). When normalised data were considered, a significant overall between-group effect was found for RER (F = 4.22, *p* = 0.027, η_p_^2^ = 0.26), highlighting a difference between dGTE and dGTE+ by week 8 (*p* = 0.034).

Whilst a significant interaction effect was also found for mean FAT_OX_ during steady state exercise (F = 3.15, *p* = 0.022, η_p_^2^ = 0.21), no significant post-hoc analyses were found, despite a +28.4% increase in steady state FAT_OX_ by week 8 with dGTE+ (*p* > 0.05). However, a significant interaction was found with relative FAT_OX_ (F = 3.98, *p* = 0.007, η_p_^2^ = 0.25), with a +54.2% increase in FAT_OX_·FFM^−1^ by week 8 compared to week 4 for dGTE+ only (*p* = 0.036). Normalised data also highlighted a significant between-group effect for FAT_OX_ by week 8 (F = 4.050, *p* = 0.031, η_p_^2^ = 0.252) with a +0.84 ± 0.48 fold change for dGTE+ compared with both PL (−0.22 ± 0.14) and dGTE (−0.22 ± 0.15). However, post-hoc analyses only revealed a trend between PL and dGTE (*p* = 0.064) and dGTE and dGTE+ (*p* = 0.063). A similar pattern was found for normalised FAT_OX_·FFM^−1^ (F = 3.93, *p* = 0.033, η_p_^2^ = 0.25), with no significant post-hoc analyses (PL compared with dGTE, *p* = 0.071; dGTE compared with dGTE+, *p* = 0.068).

No significant effects were found for mean carbohydrate oxidation stability, despite a noted -11.9% reduction by week 8 for dGTE+ (*p* > 0.05). Similarly, mean fat oxidation stability did not significantly improve with either dGTE formula, despite an observed trend (interaction effect: F = 2.36, *p* = 0.066, η_p_^2^ = 0.16) by week 8 for dGTE+. No significant differences were observed for normalised data for carbohydrate or fat oxidation stability at week 4 or 8 (*p* > 0.05). No significant differences were reported for EE within or between groups (*p* > 0.05). However, a significant interaction effect was found for both the contribution of carbohydrate (F = 4.37, *p* = 0.004, η_p_^2^ = 0.27) and the contribution of fat (F = 4.29, *p* = 0.005, η_p_^2^ = 0.26) to EE, highlighting a −17.8% reduction in carbohydrate contribution (*p* = 0.008) and a +64.8% increase in fat contribution (*p* = 0.006) to EE for dGTE at week 8 compared to week 4, respectively. When normalised data were considered, a significant between-group effect was observed for both carbohydrate (F = 3.66, *p* = 0.041, η_p_^2^ = 0.23) and fat (F = 3.73, *p* = 0.039, η_p_^2^ = 0.237) contribution to EE by week 8. However, post-hoc analyses only indicated a difference between dGTE and dGTE+ for carbohydrate contribution to EE (*p* = 0.043), but not fat contribution (*p* = 0.083).

### 3.4. Body Composition, HR and Blood Pressure

Mean body composition, resting HR and blood pressure measures are shown in Table 6. No significant differences were reported at baseline for all variables (*p* > 0.05). No significant differences were observed for body mass, BMI, body fat, fat-free mass, fat mass, WHR, SAD, VFA or blood pressure within or between groups (*p* > 0.05). A main effect was reported for waist circumference (F = 4.65, *p* = 0.014, η_p_^2^ = 0.16), with a −0.8 ± 0.2 cm relative decrease at week 4 compared to baseline for PL only (*p* = 0.019). A main effect was also reported for abdominal circumference (F = 6.19, *p* = 0.004, η_p_^2^ = 0.21), with a −1.6 ± 0.4 cm relative decrease by week 4 for dGTE compared to baseline (*p* = 0.004). Resting HR was significantly reduced by week 4 for dGTE+ compared with dGTE only (main interaction; F = 2.61, *p* = 0.047, η_p_^2^ = 0.18).

### 3.5. Blood Analytes

Mean serum cardio-metabolic markers, including normalised fold change, are shown in Table 7 and Table 8. No significant differences were reported at baseline for all variables (*p* > 0.05), except adiponectin. Mean adiponectin levels for dGTE+ were significantly greater compared to PL at baseline (*p* = 0.036), week 4 (*p* = 0.021) and week 8 (*p* = 0.049). No other main effects were found for all variables, except for LDL-c, where a main interaction effect was observed (F = 2.75, *p* = 0.039, η_p_^2^ = 0.19). However, post-hoc analyses did not reveal any significant differences for LDL-c within or between groups despite a relative −0.38 ± 0.22 mmol·L^−1^ reduction for dGTE+, and a relative +0.30 ± 0.12 mmol·L^−1^ increase for dGTE over the intervention. When data were normalised, a significant interaction effect was found for LDL-c over the intervention (F = 3.95, *p* = 0.033, η_p_^2^ = 0.25) highlighting the difference between dGTE and dGTE+ only (*p* = 0.038).

Mean serum liver function markers, including normalised fold change, are shown in Table 9 and Table 10. All mean data were considered within normal reference ranges for all variables. No significant effects were found for ALT, AST or bilirubin, despite relative reductions of −16.22 ±11.52 U·L^−1^, −8.22 ± 7.43 U·L^−1^ and −0.89 ± 1.78 µmol·L^−1^, respectively, by week 8 for PL (*p* > 0.05). However, ALP significantly reduced by −6.67 ± 3.03 U·L^−1^ over time (F = 6.63, *p* = 0.003, η_p_^2^ = 0.22) for PL only (*p* ≤ 0.025 for both baseline and week 4 compared with week 8). When data were normalised, a significant between-group effect was reported overall for ALT only (F = 4.88, *p* = 0.017, η_p_^2^ = 0.29), with the highlighted difference between PL and dGTE+ (*p* = 0.015) likely explained by the relative decrease for ALT in PL over time. All liver enzymes, and bilirubin, were unaffected by both dGTE and dGTE+ supplementation.

## 4. Discussion

This study aimed to assess the longer-term impact of moderate dGTE supplementation (with or without antioxidant nutrients) on fat oxidation, body composition and cardio-metabolic risk factors in overweight, but healthy individuals engaged in regular exercise. The findings from the current study demonstrated that a moderate dose of dGTE consumed over 8 weeks did not result in a statistically significant change on fat oxidation rates, but did produce a non-significant increase in both MFO (+12.7%) and relative MFO (+13.9%). In contrast, the use of a novel dGTE+ formula, containing antioxidants in addition to dGTE, had a significant impact (moderate–large effect size) on MFO (+45.5%) and relative MFO (+49.4%) by week 8 of supplementation in recreationally active, overweight individuals. Additionally, a large effect size for FAT_MIN_ (+22.5%) was observed with dGTE+ over the last 4 weeks of this study. Metabolic changes were also observed by week 8 for dGTE+, with a large effect size reported for improvements in steady state substrate utilisation, evidenced by lower mean RER (−5.3%), improved relative FAT_OX_ (+55.2%) and increased the contribution of fat to total EE (+64.8%). Collectively, these findings suggest that a novel dGTE+ formula positively influenced fat oxidation in overweight individuals, particularly in the final 4 weeks of this study.

With regards to the methodological approaches to FAT_OX_ measures, concerns have been noted in the literature pertinent to reliability and variability [53,54,55]. This was also observed in the current study with dGTE+ improving steady state FAT_OX_ by +28.4%, which was comparable to previous work undertaken [25], despite being non-significant. Whilst mean MFO and steady state FAT_OX_ rates in the current study were comparable to previous research with untrained [56], active [57,58,59] or overweight individuals [58,60,61,62], a large inter-individual range was noted (MFO range: 26–353 mg·min^−1^). As such, normalised data were also assessed to confirm or support findings. When data were normalised, taking into consideration individual responses to the intervention, MFO, relative MFO and FAT_MIN_ were all significantly enhanced with dGTE+ overall compared with PL, but not dGTE. However, for steady state exercise, normalised data only confirmed improved substrate utilisation for dGTE+ based on a reduced RER and the contribution of carbohydrate to total EE compared with dGTE. Whilst this further supports the contention that dGTE+ improved fat oxidation dynamics and substrate utilisation efficiency, data should be interpreted with caution based on individual ‘responders’ to the protocol. Overall FAT_MAX_ was not improved with dGTE+, despite a +24% increase in the latter 4 weeks of this study. This may be explained with the wide range of FAT_MAX_ values observed across this study (range: 24.1 to 52.4% $\dot{V}$O_2peak_).

Previous studies have reported mixed findings on the effect of GTE on fat oxidation, with several observing improvements in whole-body fat oxidation rates (+17–35% improvement employing a dose range of 270–400 mg·d^−1^ EGCG) [11,17,18,19,25] and no or small effects elsewhere (<+5–7% improvement with a dose range of 180–624 mg·d^−1^ EGCG) [21,22,23,24,26,63]. Indeed, a recent meta-analysis [64] highlighted a non-significant effect of EGCG supplementation on fat oxidation when compared with placebo (mean difference: 210 mg·min^−1^), although these findings were based on pooled data from only two randomised controlled trials and should be interpreted with caution. Interestingly, beneficial effects of GTE have been mainly observed with healthy, active, untrained or sedentary individuals using acute (<2 days) moderate EGCG dose [11,17,18,19]. However, within these studies, it is unclear whether enhanced fat oxidation was due to accumulated GTE intake or dose consumed prior to exercise testing considering plasma EGCG concentrations peak at ~90 mins, with a reported half-life of ~10 hours [65,66]. It is further unclear whether these short-term studies have meaningful impact in the longer term. Chronic intake of dGTE, with comparable EGCG dose, but not consumed on the day of testing, has been shown to enhance fat oxidation and exercise performance in healthy, recreationally active males [25]. However, the use of dGTE has been disputed elsewhere [26] with physically active males, and appears to offer little benefit to trained individuals in terms of substrate utilisation and/or endurance exercise performance [21,22,23,24].

Collectively, these studies indicate that EGCG (both caffeinated and decaffeinated) may be effective in recreationally active or less trained individuals, potentially via thermogenic mechanisms pertinent to COMT inhibition and increased lipolytic activity leading to enhanced FAT_OX_ during exercise [9,14], although supporting evidence to confirm this is currently lacking. In the current study, it is noteworthy that normalised FFAs were greater for both dGTE groups compared with PL, albeit non-significantly. In the longer term, the effectiveness of this mechanism may be related to COMT genotype [67] and/or specific adaptations in mitochondrial efficiency with regular EGCG consumption (particularly when coupled with exercise training). It has been proposed that EGCG consumption may act in a ‘calorie-restriction-mimetic’ manner through AMPK and sirtuin pathways. This may further modulate PGC1α, peroxisome proliferator-activated receptors (PPARs) and Forkhead box O (FOXO) gene expression, particularly in liver, adipose and skeletal muscle tissue [14,15,68]. However, this has been disputed elsewhere in animal studies, with inference that adaptations in fatty acid translocase/CD36mRNA are more likely associated with improvements in fat oxidation [69]. Future studies should aim to explore underlying mechanisms pertinent to fat metabolism gene expression, β-oxidation enzymes and fatty acid transporters following regular EGCG consumption. Further attention towards EGCG dose, as well as the effects of other potential calorie-restriction-mimetic nutrients (e.g., resveratrol, LA, curcumin, and other plant polyphenols [i.e., gallic acid]) on mitochondrial efficiency are also warranted.

In the current study, the specific use of dGTE did not significantly enhance fat oxidation measures in agreement with previous research [21,22,26,63]. However, the use of a novel dGTE+ formula does support findings particularly pertinent to less trained and/or overweight individuals [11,17,19]. The inclusion of quercetin within the dGTE+ may have reduced degradation or oxidation of EGCG within the small intestine (especially considering dietary quercetin intakes were consistent between groups across the intervention). It has been suggested that this could enhance EGCG bioavailability either directly, or through increased EGCG delivery to the large intestine, microbiota conversion to EGCG ring-fission metabolites and phenolic compounds, and subsequent elevated plasma free and conjugated forms of EGCG [31,32,65,70]. Additionally, the increased bioavailability of quercetin, even at a low dose may have supported antioxidant mechanisms of EGCG pertinent to longer-term mitochondria efficiency [70].

The additional inclusion of LA in the dGTE+ formula may have also enhanced EGCG stability through local antioxidant mechanisms, leading to increased EGCG availability. However, as EGCG bioavailability was not ascertained in the current study, this cannot be confirmed. As the use of dGTE only did not significantly influence FAT_OX_, it is feasible the observed metabolic benefits associated with dGTE+ may be specific to LA. Whilst numerous mechanisms have been proposed, LA supports mitochondrial efficiency as a cofactor for α-ketoacid dehydrogenases [71]. Reduced LA (DHLA) has also been implicated as a powerful natural antioxidant, highlighting that LA may support endogenous antioxidant status and mitochondrial protection [71,72,73]. Additionally, LA has been proposed to influence AMPK pathways which may modulate muscle glucose uptake via GLUT4 translocation, and insulin sensitivity [71,72]. Therefore, in the longer term, it is feasible that, coupled with regular exercise training, LA could support enhanced mitochondrial efficiency, FAT_OX_ and subsequent weight loss. However, whilst a recent meta-analysis supports the contention that LA may elicit small, meaningful weight reduction benefits, studies ranging from 8–52 weeks have employed higher LA doses between 300 and 1800 mg·d^−1^ [74], which may explain such findings. As a lower dose of LA was employed in the current study, it is proposed that the synergistic inclusion of LA and quercetin may have acted to enhance EGCG bioavailability leading to up-regulated fat oxidation pathways in addition to regular exercise. Further research is warranted to confirm EGCG bioavailability with synergistic inclusion of quercetin and/or LA, along with cellular mechanistic pathways, particularly considering higher doses commonly used in commercial formulas.

Whilst the use of dGTE+ impacted on fat oxidation, by the end of the intervention, neither dGTE supplementation influenced overall body composition measures. This was despite relative, albeit small reductions in overall mean body mass (−0.50 kg), body fat percentage (−0.50%), fat mass (−0.37 kg), abdominal circumference (−0.77 cm) or SAD (−0.61 cm), particularly for dGTE+ compared with PL. Furthermore, the relative reduction in estimated VFA with dGTE+ was ~3-fold greater than for both dGTE and PL, albeit non-significant. Maintenance of habitual exercise only marginally impacted on body composition indices as evidenced by a significant reduction in waist and abdomen circumference for PL and dGTE at week 4 within group only. Collectively, the results indicate that when habitual exercise and dietary intake are controlled for, dGTE supplementation did not favour improved body composition indices. As dGTE+ only enhanced FAT_OX_ by week 8, any changes in body composition attributed to EGCG mechanisms may require longer interventions based on overall relative patterns in body composition indices with dGTE+. Indeed, a previous meta-analysis [9] highlighted that GT consumption across studies exceeding 12 weeks resulted in a small, positive effect on body mass loss (−1.31 kg), with greater effects (−1.60 kg) observed for low habitual caffeine intake users.

However, the lack of efficacy of dGTE to modify body composition measures does concur with other longer-term studies (EGCG dose: 199–843 mg·d^−1^) [28,67,75], although physical activity was only partially monitored in these studies, and one study [28] also introduced a low energy diet as part of the intervention. Elsewhere, significant improvements in body composition have been reported with GTE consumption (EGCG dose: 100–225 mg·d^−1^) [12,13,29], particularly when combined with an exercise intervention in previously sedentary individuals [12,29]. Interestingly, in one study [29] exercise prescription began at 40% HR reserve (HRR) and progressed up to 50-59% HRR. Other studies utilising training at FAT_MAX_ (range:~34–52% $\dot{V}$O_2max_ [62,76,77]) in overweight, obese or diabetic women demonstrated significant improvements in body mass, BMI, fat mass and visceral trunk fat. In the current study, physical activity guidelines (>150 mins·wk^−1^ at a moderate intensity ~60–65% $\dot{V}$O_2peak_ in line with other studies [18,78]) were maintained. As this intensity was above participants’ FAT_MAX_, this likely favoured carbohydrate metabolism and, in part, may explain the small decrease in MFO observed with PL. Combining dGTE+ with individualised FAT_MAX_ training in overweight individuals may therefore favourably improve body composition, warranting further research. 

Cardio-metabolic variables were largely unaffected by either dGTE supplementation, with the exception of a medium effect size reported for LDL-c which was ~11% lower with dGTE+ by week 8, and further supported via normalised values. The relative reduction in total LDL-c across the intervention with dGTE+ (−0.38 ± 0.22 mmol·L^−1^) was comparable to previous studies [5,79], which may be important as the mean values were considered elevated. This concurs with a recent meta-analysis indicating that LDL-c is likely reduced with regular green tea consumption (all sources) by −0.12 mmol·L^−1^ [80], and likely further reduced (−0.14 mmol·L^−1^) when capsulated or decaffeinated green tea products are used [80]. Interestingly, the observed effects of green tea consumption (all sources) appear greater over time, with noted reductions for total cholesterol (−0.14 mmol·L^−1^) and triglycerides (−0.23 mmol·L^−1^) in longer-term clinical trials (>12 weeks) [80]. In the current study, as cholesterol biomarkers were largely unaffected with dGTE alone, this infers that the observed reductions in LDL-c with dGTE+ may have been attributed to either increased bioavailability via inclusion of low-dose quercetin, or separately via the inclusion of LA acting independently. Indeed, other reviews [81] have reported significant reductions in LDL-c of −0.33 mmol·L^−1^ with LA alone, which is comparable to that observed in the current study. 

Both leptin and adiponectin levels were unaffected across the intervention with dGTE or dGTE+. This agrees with a recent meta-analysis [82] demonstrating that leptin concentration is likely unaffected with GT (from all sources) when consumed for less than 12 weeks. In contrast, longer-term use of GT (>12 weeks) likely increases leptin concentrations (effect size: 2.90 [82]). As appetite hormones and body composition changes were largely unaffected by either dGTE in the current study, this suggests that observed benefits of dGTE+ on fat oxidation by week 8 may have been attributed to gene expression associated with mitochondrial efficiency [14]. It is noteworthy that adiponectin levels were significantly higher with dGTE+ compared to PL at baseline, week 4 and 8. Adiponectin has been associated with synergistic activation of AMPK, p38 mitogen-activated protein kinase (MAPK) and PPARα in skeletal muscle, and hence fatty acid oxidation [83]. Higher adiponectin levels, combined with dGTE+, may have favourably altered AMPK kinetics, resulting in enhanced mitochondrial fatty acid oxidation across the intervention. In accordance with previous reviews [82], it is feasible that longer-term dGTE consumption further influences appetite regulation favouring improved body composition characteristics, especially when combined with enhanced fat metabolism. 

It is also noteworthy, when considering normalised values, that mean insulin, HOMA-IR and leptin changes were elevated with PL across the intervention (albeit non-significantly), with negligible changes observed for dGTE or dGTE+. It is therefore unclear whether the small changes observed for PL were related to dietary composition differences, or whether dGTE acted in a ‘protective’ manner. However, collectively, these results support previous findings that GTE was ineffective in improving cardio-metabolic function [6,27,28], but differ from other studies where positive effects of GTE have been observed with [30] or without exercise [5,13]. Interestingly, one other study [84] employed a theaflavin-enriched GTE strategy, resulting in significant reductions in LDL-c and total cholesterol. This combination was proposed to reduce intestinal absorption and/or increased excretion of cholesterol, as well as LDL-c liver receptor up-regulation. The combined use of quercetin and LA with dGTE+ in the current study may have similarly enhanced dGTE effectiveness. 

Whilst dGTE+ also resulted in a significant reduction in resting HR by week 4 compared to dGTE, but not by week 8, this did not impact on blood pressure measurements. This is somewhat surprising as a recent meta-analysis on green tea (from all sources) highlighted studies (≤12 weeks) resulting in a reduction of −1.32 mmHg for SBP (non-significant) and -1.78 mmHg for DBP (deemed significant), with lower observed effects for capsulated green tea, and green tea (all sources) consumed over longer periods (> 12 weeks) [85]. In the current study, dGTE+ resulted in a reduction of −3.33 mmHg (for SBP) and -1.78 mmHg (for DBP), which exceeded the observed reduction expected for exercise/placebo (−2.44 mmHg for SBP and −0.78 mmHg for DBP), but was deemed non-significant. Whilst this may be partly explained by inter-individual variance in blood pressure responses, our results suggest that decaffeinated green tea extract did not impact on blood pressure response above that expected through maintenance of regular exercise. 

An important aspect of the current study was the inclusion of liver enzyme and bilirubin measures. This was undertaken based on current concerns with the potential for GTE-induced hepatotoxicity [86]. However, no significant elevations were observed for any measures, indicating relative safety of dGTE at a moderate dose across the intervention period. Whilst it has been noted that fasted intake may be a contributing factor to GTE-induced heptatoxicity [87], other studies have highlighted the potential for reduced bioavailability when GTE is consumed with food [20]. In the current study, participants were requested to consume dGTE away from food in a split-dose manner. As such, it is possible that consumption may have occurred in an absorptive state. Coupled with a lower single dose per consumption (i.e., 200 mg EGCG per capsule), this may, in part, explain normal liver function observed in the current study. Whilst this supports previous research [6], and is in line with current recommendations [87,88], the efficacy of lower doses is warranted, as well as awareness of product composition. As example, unpublished data from our laboratory indicate that exposure to the specific dGTE used in this study impacts AMPK activity and mitochondrial oxidative stress to improve survival of hepatocytes in vitro. Therefore, due consideration of the effect of dGTE on oxidative stress pathways at a cellular level is also needed to understand the molecular mechanisms impacting MFO, as other products may differ in terms of biological effects. 

It is important to note several limitations of the current study. As previously mentioned, EGCG bioavailability was not undertaken, and as no LA-specific intervention was included, findings should be interpreted with caution. Further studies investigating the specific effects of LA with or without dGTE are therefore warranted. In the current study a standardised dGTE (70% EGCG) product was sourced based on our previous research [25]. Whilst this was independently verified, the content of other catechins (e.g., (−)-epicatechin-3-gallate (ECG)), flavonols (e.g., isoquercitin), flavonol glycosides and their aglycones, were not quantified. Whilst EGCG is the most abundant catechin within dGTE, the remaining catechin/flavonol compounds may have contributed to findings. Consideration to the presence, and biological activity, of other catechins/flavonols within dGTE supplementation is therefore warranted in future research. 

In a similar context, consideration to dietary consistency and total polyphenol intake is also warranted. In the present study, relative dietary intake consistency was established in the 7 day period leading into each testing session. Furthermore, estimated quercetin intake (overall mean: 20.6 ± 8.5 mg·d^−1^) was comparable between groups, and similar to previously reported research [89,90]. Therefore, any influence of the dGTE+ compound may have related to the enhanced bioavailability of EGCG with additional quercetin. However, it is feasible that individual dietary variance and total polyphenol intake outside of collection periods may have limited findings. Future research should therefore consider the impact of dGTE strategies in conjunction with low or high dietary polyphenol intake when combined with exercise strategies. Similarly, whilst exercise was monitored across the full intervention period, we did not investigate other components of physical activity such as non-exercise activity thermogenesis or sedentary periods which could affect overall metabolic flexibility and/or fat oxidation, as previously reported [91,92], even when physical activity guidelines are met. Finally, although testing sessions were standardised, as the majority of the participants were females, it is feasible that hormonal variance pertinent to menstrual cycle regulation may have influenced metabolic changes. However, this has been disputed elsewhere [93].

## 5. Conclusions

The use of a novel dGTE+ (containing quercetin and LA) was effective in improving MFO and FAT_MIN_ in healthy, overweight individuals by week 8 of the intervention. This corresponded with improved FAT_OX_ during steady state exercise and the reduced contribution of carbohydrate to total energy expenditure. However, when habitual exercise and dietary intake are maintained, this did not translate to improved body composition or markers of cardio-metabolic health, with the exception of reduced LDL-c. Future studies investigating dose–response and longer-term implications of dGTE+, particularly in combination with dietary strategies to support weight-loss in overweight individuals are warranted.

## Figures and Tables

**Table 1:** Baseline characteristics for intervention groups.

	PL	dGTE	dGTE+
Variable	(*n* = 9; 4 male, 5 female)	(*n* = 9; 2 male, 7 female)	(*n* = 9; 1 male, 8 female)
Age (years)	35 ± 7 *	46 ± 5	47 ± 5
Height (m)	1.71 ± 0.04	1.66 ± 0.06	1.67 ± 0.07
Body mass (kg)	82.7 ± 13.0	74.3 ± 7.4	75.6 ± 9.4
BMI (kg·m^2^)	28.2 ± 4.1	26.8 ± 2.4	27.1 ± 2.3
Body fat (%)	32.0 ± 7.9	33.2 ± 6.6	36.3 ± 6.2
$\dot{V}$O_2peak_ (L·min^−1^)	2.5 ± 0.7	2.4 ± 0.6	2.1 ± 0.5
$\dot{V}$O_2peak_ (mL·kg^−1^·min^−1^)	30.3 ± 6.3	32.4 ± 5.2	27.8 ± 5.6

Data presented as the mean ± SD. PL = placebo; dGTE = decaffeinated green tea extract; dGTE+ = decaffeinated green tea extract with antioxidants; BMI = body mass index. No significant differences reported between groups, except age. * denotes significant difference (*p* ≤ 0.002) to dGTE and dGTE+.

**Table 2:** Mean relative energy and macronutrient intake comparisons between groups.

Variable	PL	dGTE	dGTE+
**Energy intake (kcal·kg^−1^·day^−1^)**			
Baseline	23.7 ± 1.2	25.8 ± 1.4	25.4 ± 1.3
Week 4	24.8 ± 1.8	25.4 ± 1.6	26.1 ± 1.7
Week 8	24.4 ± 1.5	25.5 ± 1.3	25.6 ± 1.6
**Fat (g·kg^−1^·day^−1^)**			
Baseline	0.9 ± 0.1	0.9 ± 0.1	1.0 ± 0.1
Week 4	1.0 ± 0.1	0.8 ± 0.1	1.0 ± 0.1
Week 8	0.9 ± 0.1	0.9 ± 0.1	1.0 ± 0.1
**Carbohydrate (g·kg^−1^·day^−1^*)***			
Baseline	2.8 ± 0.2	3.0 ± 0.2	2.7 ± 0.2
Week 4	2.7 ± 0.3	3.1 ± 0.2	2.8 ± 0.3
Week 8	2.7 ± 0.2	3.0 ± 0.2	2.7 ± 0.2
**Protein (g·kg^−1^·day^−1^)**			
Baseline	0.9 ± 0.0	1.2 ± 0.1	1.1 ± 0.1
Week 4	0.9 ± 0.1	1.2 ± 0.2	1.1 ± 0.1
Week 8	1.0 ± 0.1	1.1 ± 0.1	1.1 ± 0.1

Data represent average daily intake. PL = placebo; dGTE = decaffeinated green tea extract; dGTE+ = decaffeinated green tea extract with antioxidants. No significant differences reported between or within groups (*p* > 0.05).

**Table 3:** Estimated mean training load, monotony and strain between intervention groups prior to and across the experimental period.

Variable	PL	dGTE	dGTE+
**Weekly training load (AU)**			
Baseline	869 ± 129	1134 ± 136	848 ± 144
Week 4	996 ± 139	893 ± 127	918 ± 96
Week 8	888 ± 166	850 ± 99	795 ± 141
**Training monotony (AU)**			
Baseline	0.85 ± 0.08	0.92 ± 0.12	0.90 ± 0.07
Week 4	0.82 ± 0.05	0.89 ± 0.07	1.02 ± 0.09
Week 8	0.78 ± 0.02	0.92 ± 0.08	0.98 ± 0.16
**Training strain (AU)**			
Baseline	764 ± 139	1090 ± 205	819 ± 223
Week 4	787 ± 88	824 ± 143	1070 ± 188
Week 8	666 ± 116	804 ± 137	969 ± 350

Data represent arbitrary units (AU). PL = placebo; dGTE = decaffeinated green tea extract; dGTE+ = decaffeinated green tea extract with antioxidants. No significant differences reported between or within groups (*p* > 0.05).

**Table 4:** Mean fat oxidation measures at baseline (week 0), week 4 and 8 between intervention groups.

	PL			dGTE			dGTE+		
Variable	Baseline	Week 4	Week 8	Baseline	Week 4	Week 8	Baseline	Week 4	Week 8
MFO (mg·min^−1^)	228.1 ± 23.4	219.5 ± 34.1	201.1 ± 25.2	199.0 ± 30.8	223.2 ± 32.3	224.4 ± 27.8	154.4 ± 20.6	176.6 ± 19.3	224.6 ± 23.2 *
MFO·FFM^−1^ (mg·min^−1^)	4.7 ± 0.7	4.1 ± 0.6	3.7 ± 0.5	4.0 ± 0.6	4.4 ± 0.6	4.5 ± 0.5	3.2 ± 0.4	3.7 ± 0.4	4.7 ± 0.5 *
FAT_MAX_ (% $\dot{V}$O_2peak_)	35.2 ± 2.0	38.6 ± 2.2	38.0 ± 1.9	36.5 ± 6.0	34.1 ± 1.8	36.5 ± 2.9	39.5 ± 2.6	34.4 ± 1.3	42.7 ± 2.3
HR at FAT_MAX_ (%HR_max_)	57.6 ± 1.9	58.5 ± 2.1	57.7 ± 1.7	56.3 ± 1.5	57.9 ± 1.6	56.8 ± 1.7	57.1 ± 1.7	57.5 ± 2.1	58.1 ± 1.6
FAT_MIN_ (% $\dot{V}$O_2peak_)	61.7 ± 4.3	64.7 ± 4.2	56.1 ± 4.0	60.1 ± 4.1	54.9 ± 3.4	57.2 ± 3.6	58.3 ± 2.6	55.1 ± 1.9	67.6 ± 3.6 ^#^

PL = placebo; dGTE = decaffeinated green tea extract; dGTE+ = decaffeinated green tea extract with antioxidants. MFO = maximal fat oxidation rate; MFO·FFM^−1^ = relative maximal fat oxidation rate (per kg FFM); FAT_MAX_= exercise intensity at which MFO occurs; FAT_MAX_ HR = heart rate at MFO expressed relative to maximum HR; FAT_MIN_ = exercise intensity where FAT_OX_ deemed zero. * significantly different from baseline within group (*p* ≤ 0.024); # significantly different to week 4 within group (*p* = 0.003).

**Table 5:** Mean steady state exercise measures at baseline (week 0), week 4 and 8 between intervention groups.

	PL			dGTE			dGTE+		
Variable	Baseline	Week 4	Week 8	Baseline	Week 4	Week 8	Baseline	Week 4	Week 8
RER	0.91 ± 0.01	0.90 ± 0.02	0.92 ± 0.02	0.90 ± 0.02	0.92 ± 0.02	0.93 ± 0.01	0.92 ± 0.01	0.94 ± 0.01	0.89 ± 0.01 *
FAT_OX_ (mg·min^−1^)	224.4 ± 27.7	237.3 ± 48.9	181.6 ± 36.8	222.3 ± 41.4	185.7 ± 41.1	161.3 ± 31.1	169.1 ± 37.6	152.6 ± 45.8	217.2 ± 31.7
FAT_OX_·FFM^−1^ (mg·min^−1^)	4.1 ± 0.5	4.4 ± 0.9	3.3 ± 0.6	4.5 ± 0.8	3.6 ± 0.7	3.2 ± 0.6	3.4 ± 0.7	2.9 ± 0.7	4.5 ± 0.7 *
CHO_OX_ST (%)	62.6 ± 5.5	61.2 ± 5.2	68.2 ± 4.1	64.4 ± 3.9	67.6 ± 4.8	63.5 ± 5.3	72.0 ± 2.8	71.7 ± 2.1	63.1 ± 4.2
FAT_OX_ST (%)	31.5 ± 5.0	35.4 ± 7.5	28.3 ± 6.0	34.4 ± 7.4	32.2 ± 5.7	26.5 ± 5.5	27.8 ± 5.5	22.5 ± 4.6	36.8 ± 5.3
Total EE (kcal.hr^−1^)	446.6 ± 27.6	420.4 ± 25.8	435.8 ± 27.1	421.0 ± 30.4	407.5 ± 31.8	404.4 ± 29.0	377.9 ± 28.9	383.3 ± 39.4	374.4 ± 30.3
CHOcont. EE (%)	69.9 ± 4.0	67.1 ± 6.9	75.2 ± 5.2	69.1 ± 6.0	74.3 ± 5.8	77.4 ± 4.6	75.0 ± 4.6	79.7 ± 4.5	65.5 ± 4.6 *
FATcont. EE (%)	30.3 ± 4.1	33.4 ± 6.8	25.3 ± 5.1	31.4 ± 5.8	26.5 ± 5.4	23.3 ± 4.4	25.0 ± 4.6	21.0 ± 4.1	34.6 ± 4.7 *

PL = placebo; dGTE = decaffeinated green tea extract; dGTE+ = decaffeinated green tea extract with antioxidants. RER = respiratory exchange ratio; FAT_OX_ = fat oxidation rate; FAT_OX_·FFM^−1^ = relative fat oxidation rate (per kg FFM); CHO_OX_ST= carbohydrate oxidation stability (expressed as %); FAT_OX_ST = fat oxidation stability (expressed as %); EE = energy expenditure; CHOcont.EE = contribution of carbohydrate to total EE; FATcont.EE = contribution of fat to total EE. * significantly different to week 4 within condition (*p* ≤ 0.036).

**Table 6:** Body composition, resting heart rate and blood pressure measures at baseline (week 0), week 4 and 8 between intervention groups.

	PL			dGTE			dGTE+		
Variable	Baseline	Week 4	Week 8	Baseline	Week 4	Week 8	Baseline	Week 4	Week 8
Body mass (kg)	82.7 ± 4.3	82.5 ± 4.3	82.6 ± 4.2	74.3 ± 2.5	73.8 ± 2.3	73.9 ± 2.4	75.6 ± 3.1	75.3 ± 3.2	75.1 ± 3.1
BMI (kg·m^2^)	28.2 ± 1.4	28.1 ± 1.4	28.2 ± 1.3	26.8 ± 0.8	26.7 ± 0.9	26.7 ± 0.9	27.1 ± 0.8	27.1 ± 0.8	27.0 ± 0.9
Body fat (%)	32.0 ± 2.6	31.7 ± 2.5	32.2 ± 2.7	33.2 ± 2.2	32.7 ± 2.3	32.9 ± 2.2	36.3 ± 2.1	36.1 ± 2.1	35.8 ± 2.2
FFM (kg)	56.1 ± 3.4	55.9 ± 3.2	55.5 ± 3.1	49.6 ± 2.5	49.7 ± 2.5	49.5 ± 2.4	48.0 ± 2.5	48.0 ± 2.5	48.2 ± 2.4
FM (kg)	26.6 ± 2.8	26.3 ± 2.7	26.7 ± 2.8	24.6 ± 1.8	24.1 ± 1.9	24.3 ± 1.8	27.3 ± 2.1	27.3 ± 2.3	27.0 ± 2.4
WHR	0.84 ± 0.03	0.83 ± 0.02	0.83 ± 0.03	0.82 ± 0.02	0.82 ± 0.02	0.83 ± 0.02	0.80 ± 0.01	0.80 ± 0.01	0.80 ± 0.01
Waist C (cm)	89.5 ± 3.2	88.7 ± 3.1 *	89.0 ± 3.0	85.7 ± 2.4	85.3 ± 2.5	85.8 ± 2.6	85.2 ± 2.0	84.9 ± 1.9	84.9 ± 2.0
Ab. C (cm)	102.0 ± 2.3	101.8 ± 2.6	101.9 ± 2.6	100.8 ± 2.8	99.2 ± 2.7 *	99.7 ± 2.8	100.9 ± 2.9	100.4 ± 2.9	100.1 ± 2.9
SAD (cm)	23.3 ± 0.9	23.1 ± 0.9	23.0 ± 0.9	21.6 ± 0.5	21.1 ± 0.7	21.3 ± 0.7	21.9 ± 0.7	21.6 ± 0.7	21.3 ± 0.8
VFA (cm^2^)	1740.1 ± 140.8	1739.3 ± 151.8	1714.8 ± 135.7	1576.0 ± 116.1	1554.7 ± 134.4	1550.1 ± 151.1	1778.4 ± 143.8	1748.3 ± 136.6	1699.6 ± 161.7
HR (b·min^−1^)	68 ± 3	65 ± 2	69 ± 4	62 ± 3	68 ± 4	65 ± 2	63 ± 2	58 ± 1 #	60 ± 2
SBP (mmHg)	130 ± 9	128 ± 9	128 ± 8	117 ± 3	120 ± 4	115 ± 4	114 ± 6	113 ± 4	111 ± 4
DBP (mmHg)	84 ± 5	84 ± 6	84 ± 5	78 ± 2	78 ± 1	78 ± 2	77 ± 3	77 ± 3	75 ± 2

PL = placebo; dGTE = decaffeinated green tea extract; dGTE+ = decaffeinated green tea extract with antioxidants. BMI = body mass index; FFM = fat free mass; FM = fat mass; WHR = waist to hip ratio; waist C = waist circumference; Ab. C = abdominal circumference; SAD = sagittal abdominal diameter; VFA = estimated visceral fat area; HR = resting heart rate; SBP = systolic blood pressure; DBP = diastolic blood pressure. * significantly different to baseline within group only (*p* ≤ 0.019). # significantly different to dGTE at timepoint (*p* = 0.05).

**Table 7:** Mean serum cardio-metabolic markers at baseline (week 0), week 4 and 8 between intervention groups.

	PL			dGTE			dGTE+		
Variable	Baseline	Week 4	Week 8	Baseline	Week 4	Week 8	Baseline	Week 4	Week 8
Glucose (mmol·L^−1^)	5.36 ± 0.10	5.37 ± 0.14	5.41 ± 0.13	5.78 ± 0.17	5.53 ± 0.21	5.57 ± 0.22	5.32 ± 0.16	5.29 ± 0.13	5.23 ± 0.15
Insulin (pmol·L^−1^)	61.67 ± 12.34	66.33 ± 13.66	71.33 ± 15.38	48.44 ± 6.04	48.22 ± 5.91	43.78 ± 4.85	39.67 ± 5.47	49.00 ± 7.00	43.22 ± 7.01
%B	88.06 ± 9.87	92.13 ± 9.98	94.94 ± 9.80	66.02 ± 5.07	71.70 ± 4.05	68.57 ± 6.08	67.80 ± 5.83	79.64 ± 7.27	73.09 ± 4.82
%S	114.63 ± 23.30	100.87 ± 15.19	91.07 ± 11.66	121.32 ± 15.36	121.12 ± 14.40	132.39 ± 19.49	155.48 ± 21.10	131.66 ± 25.71	150.53 ± 26.62
HOMA-IR	1.19 ± 0.23	1.26 ± 0.26	1.35 ± 0.29	0.94 ± 0.12	0.93 ± 0.12	0.85 ± 0.09	0.76 ± 0.11	0.95 ± 0.12	0.84 ± 0.13
Leptin (ng·mL^−1^)	15.88 ± 3.83	16.69 ± 4.00	18.78 ± 3.61	17.59 ± 4.28	18.58 ± 5.39	17.46 ± 3.75	24.66 ± 5.50	24.19 ± 6.25	25.21 ± 5.89
Adiponectin (µg·mL^−1^)	5.94 ± 1.50	5.79 ± 1.47	5.82 ± 1.35	9.06 ± 1.16	8.90 ± 1.30	9.39 ± 1.45	11.17 ± 1.40 *	11.54 ± 1.37 *	11.08 ± 1.50 *
Glycerol (µmol·L^−1^)	25.44 ± 3.09	27.56 ± 3.50	27.00 ± 2.40	28.00 ± 2.70	36.44 ± 4.61	29.89 ± 3.89	31.56 ± 6.31	29.78 ± 3.42	31.11 ± 3.58
FFA (µmol·L^−1^)	310.56 ± 58.61	298.44 ± 55.71	282.56 ± 39.07	347.89 ± 51.81	436.44 ± 72.00	359.56 ± 37.09	304.22 ± 69.82	296.56 ± 36.29	287.78 ± 36.61
Chol_TOT_ (mmol·L^−1^)	5.06 ± 0.27	5.04 ± 0.21	5.17 ± 0.20	5.16 ± 0.25	5.34 ± 0.28	5.53 ± 0.33	5.31 ± 0.30	5.28 ± 0.25	5.22 ± 0.21
TG (mmol·L^−1^)	1.11 ± 0.22	1.11 ± 0.22	1.34 ± 0.30	1.03 ± 0.22	1.03 ± 0.19	1.02 ± 0.19	0.92 ± 0.13	0.74 ± 0.11	0.89 ± 0.13
HDL-c (mmol·L^−1^)	1.40 ± 0.16	1.37 ± 0.14	1.33 ± 0.13	1.65 ± 0.16	1.68 ± 0.17	1.73 ± 0.19	1.75 ± 0.15	1.82 ± 0.18	1.76 ± 0.14
LDL-c (mmol·L^−1^)	3.13 ± 0.26	3.17 ± 0.20	3.20 ± 0.16	3.04 ± 0.19	3.19 ± 0.19	3.34 ± 0.22	3.42 ± 0.28	3.12 ± 0.26	3.04 ± 0.25

PL = placebo; dGTE = decaffeinated green tea extract; dGTE+ = decaffeinated green tea extract with antioxidants. %B = Homeostasis Model Assessment (HOMA2) steady state beta cell function %; %S = HOMA2 insulin sensitivity %; HOMA-IR = HOMA2 insulin resistance; FFA = free fatty acids; Chol_TOT_ = total cholesterol; TG = triglycerides; HDL-c = high-density lipoprotein cholesterol; LDL-c = low-density lipoprotein cholesterol. * significantly different to PL at timepoint (*p* ≤ 0.049).

**Table 8:** Normalised mean fold change in serum cardio-metabolic markers by week 4 (NΔ0–4), and week 8 (NΔ0–8).

	NΔ0–4			NΔ0–8		
Variable	PL	dGTE	dGTE+	PL	dGTE	dGTE+
Glucose (mmol·L^−1^)	+0.00 ± 0.02	−0.04 ± 0.03	+0.00 ± 0.02	+0.01 ± 0.02	−0.04 ± 0.02	−0.02 ± 0.02
Insulin (pmol·L^−1^)	+0.28 ± 0.24	+0.03 ± 0.08	+0.22 ± 0.12	+0.45 ± 0.34	−0.04 ± 0.12	+0.06 ± 0.10
%B	+0.08 ± 0.09	+0.12 ± 0.08	+0.18 ± 0.08	+0.13 ± 0.12	+0.05 ± 0.07	+0.11 ± 0.07
%S	+0.01 ± 0.15	+0.03 ± 0.08	−0.16 ± 0.06	−0.07 ± 0.13	+0.16 ± 0.15	−0.05 ± 0.07
HOMA-IR	+0.17 ± 0.16	+0.02 ± 0.08	+0.26 ± 0.10	+0.29 ± 0.21	−0.02 ± 0.12	+0.09 ± 0.08
Leptin (ng·mL^−1^)	+0.13 ± 0.20	−0.02 ± 0.07	+0.00 ± 0.09	+0.26 ± 0.13	+0.02 ± 0.08	+0.02 ± 0.11
Adiponectin (µg·mL^−1^)	−0.02 ± 0.01	−0.03 ± 0.05	+0.05 ± 0.04	+0.01 ± 0.05	+0.02 ± 0.04	−0.02 ± 0.04
Glycerol (µmol·L^−1^)	+0.15 ± 0.15	+0.33 ± 0.16	+0.20 ± 0.24	+0.11 ± 0.09	+0.11 ± 0.14	+0.22 ± 0.23
FFA (µmol·L^−1^)	+0.00 ± 0.12	+0.27 ± 0.08	+0.21 ± 0.18	+0.08 ± 0.18	+0.22 ± 0.20	+0.17 ± 0.22
Chol_TOT_ (mmol·L^−1^)	+0.00 ± 0.02	+0.04 ± 0.03	+0.00 ± 0.03	+0.03 ± 0.03	+0.07 ± 0.03	−0.01 ± 0.02
TG (mmol·L^−1^)	+0.03 ± 0.11	+0.05 ± 0.11	−0.15 ± 0.11	+0.19 ± 0.16	+0.02 ± 0.09	−0.03 ± 0.06
HDL-c (mmol·L^−1^)	−0.01 ± 0.04	+0.02 ± 0.03	+0.04 ± 0.04	−0.03 ± 0.04	+0.04 ± 0.03	+0.01 ± 0.04
LDL-c (mmol·L^−1^)	+0.03 ± 0.04	+0.05 ± 0.03	−0.07 ± 0.06	+0.05 ± 0.06	+0.10 ± 0.04	−0.09 ± 0.06 *

PL = placebo; dGTE = decaffeinated green tea extract; dGTE+ = decaffeinated green tea extract with antioxidants. * denotes significantly different to dGTE (*p* = 0.038).

**Table 9:** Mean serum liver function markers at baseline (week 0), week 4 and 8 between intervention groups.

	PL			dGTE			dGTE+		
Variable	Baseline	Week 4	Week 8	Baseline	Week 4	Week 8	Baseline	Week 4	Week 8
ALT (U·L^−1^)	56.89 ± 23.14	47.22 ± 15.86	40.67 ± 11.72	34.56 ± 2.69	28.89 ± 1.62	34.22 ± 2.41	28.33 ± 1.41	30.78 ± 1.82	31.33 ± 1.85
AST (U·L^−1^)	28.33 ± 10.70	37.11 ± 15.58	20.11 ± 3.51	19.78 ± 1.23	17.11 ± 1.62	21.11 ± 1.35	19.11 ± 2.12	18.22 ± 1.42	19.33 ± 1.27
ALP (U·L^−1^)	66.89 ± 7.13 *	65.22 ± 5.96 *	60.22 ± 6.15	60.11 ± 3.36	60.00 ± 3.76	59.00 ± 4.20	59.44 ± 3.70	60.67 ± 4.18	56.67 ± 3.82
Bilirubin (µmol·L^−1^)	10.78 ± 2.26	9.33 ± 1.80	9.89 ± 1.14	9.67 ± 0.94	9.89 ± 0.92	10.00 ± 1.04	7.78 ± 0.81	7.56 ± 0.87	8.44 ± 0.67

PL = placebo; dGTE = decaffeinated green tea extract; dGTE+ = decaffeinated green tea extract with antioxidants. ALT = alanine aminotransferase; AST = aspartate transaminase; ALP = alkaline phosphatase. * significantly different to week 8 within group (*p* ≤ 0.025).

**Table 10:** Normalised mean fold change in serum liver function markers by week 4 (NΔ0–4), and week 8 (NΔ0–8).

	NΔ0–4			NΔ0–8		
Variable	PL	dGTE	dGTE+	PL	dGTE	dGTE+
ALT (U·L^−1^)	−0.06 ± 0.12	−0.15 ± 0.04	+0.11 ± 0.10	−0.15 ± 0.06	+0.01 ± 0.06	+0.11 ± 0.07 *
AST (U·L^−1^)	+0.49 ± 0.62	−0.12 ± 0.09	+0.02 ± 0.11	−0.09 ± 0.10	+0.08 ± 0.05	+0.10 ± 0.12
ALP (U·L^−1^)	−0.01 ± 0.02	+0.00 ± 0.03	+0.02 ± 0.02	−0.09 ± 0.04	−0.02 ± 0.03	−0.05 ± 0.02
Bilirubin (µmol·L^−1^)	−0.02 ± 0.14	+0.07 ± 0.13	+0.02 ± 0.11	+0.08 ± 0.15	+0.09 ± 0.15	+0.14 ± 0.09

PL = placebo; dGTE = decaffeinated green tea extract; dGTE+ = decaffeinated green tea extract with antioxidants. * denotes significantly different to PL (*p* = 0.015).

## Data Availability

The data presented in this study are available on request from the corresponding author. The data are not publicly available due to ethical considerations, in accordance with consent provided by participants on the use of confidential data.
